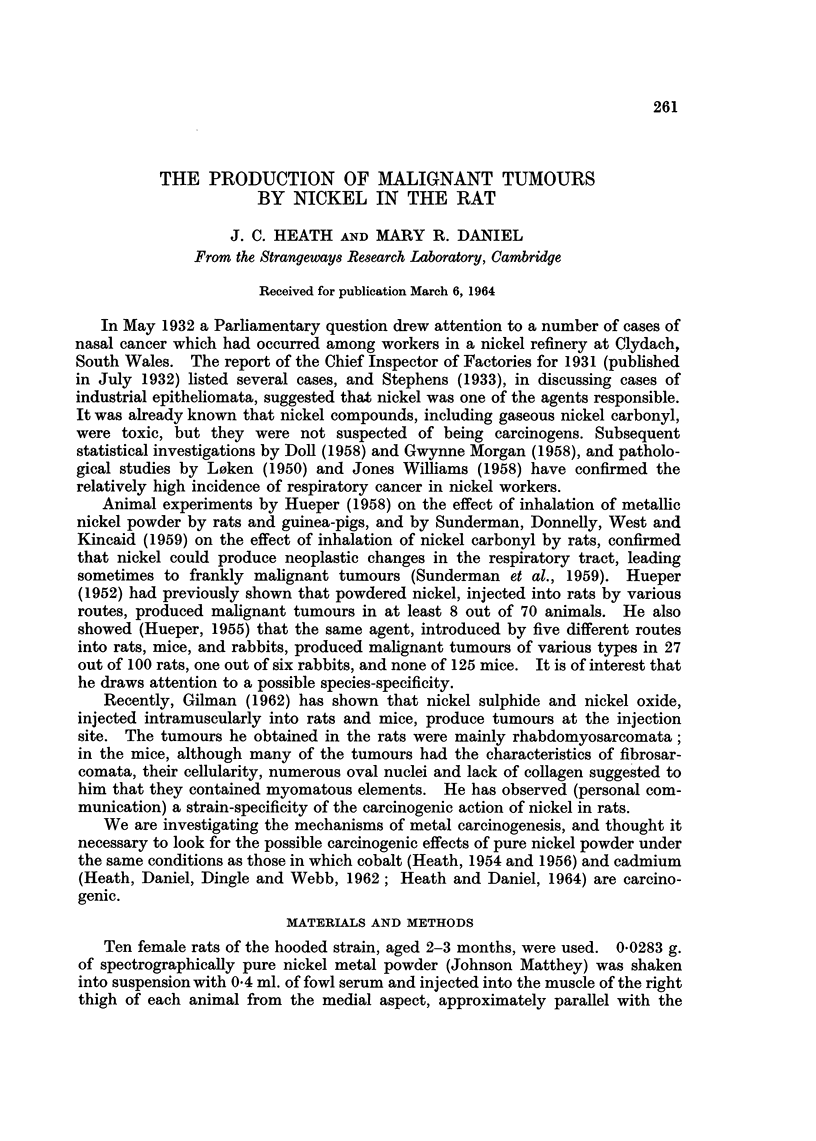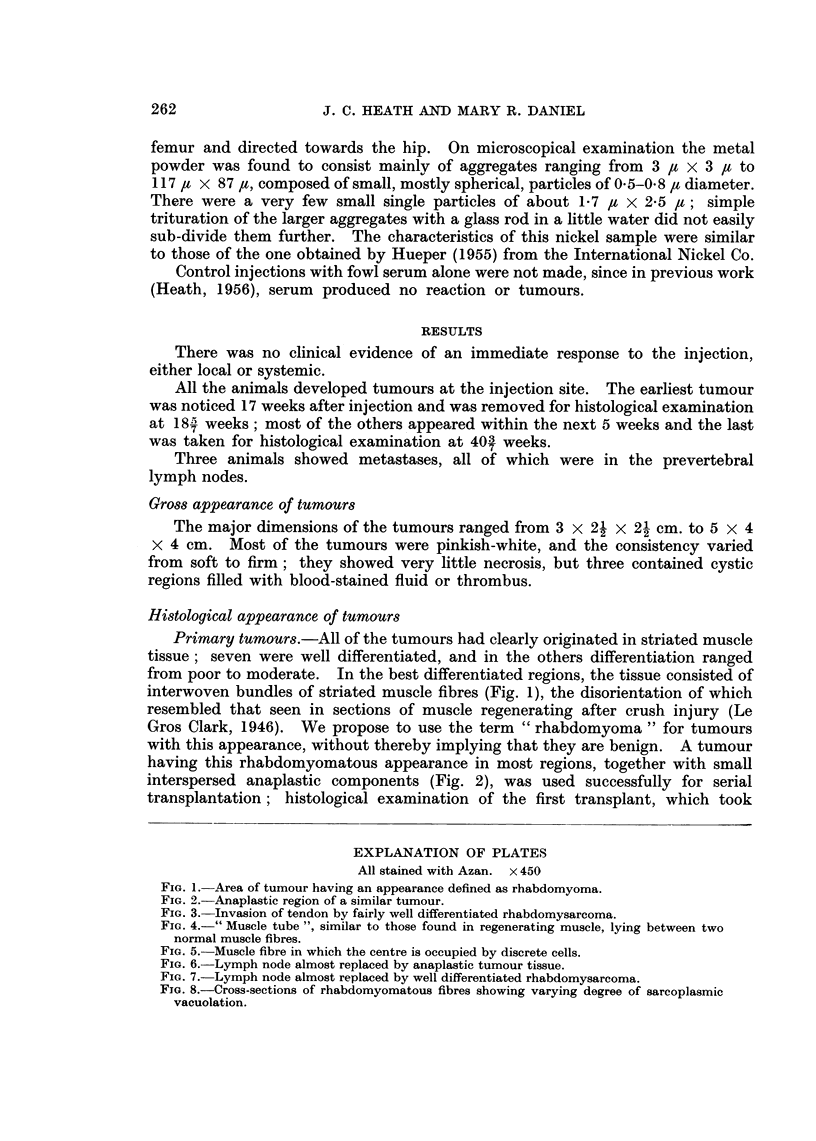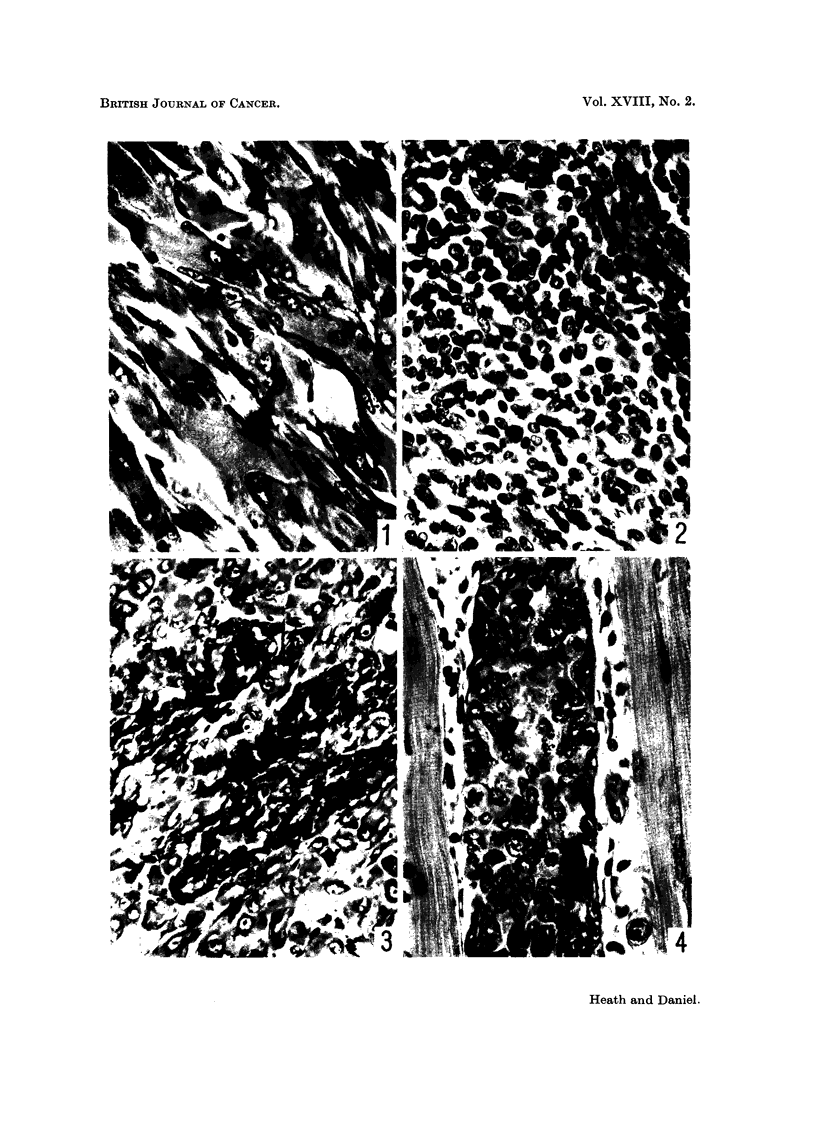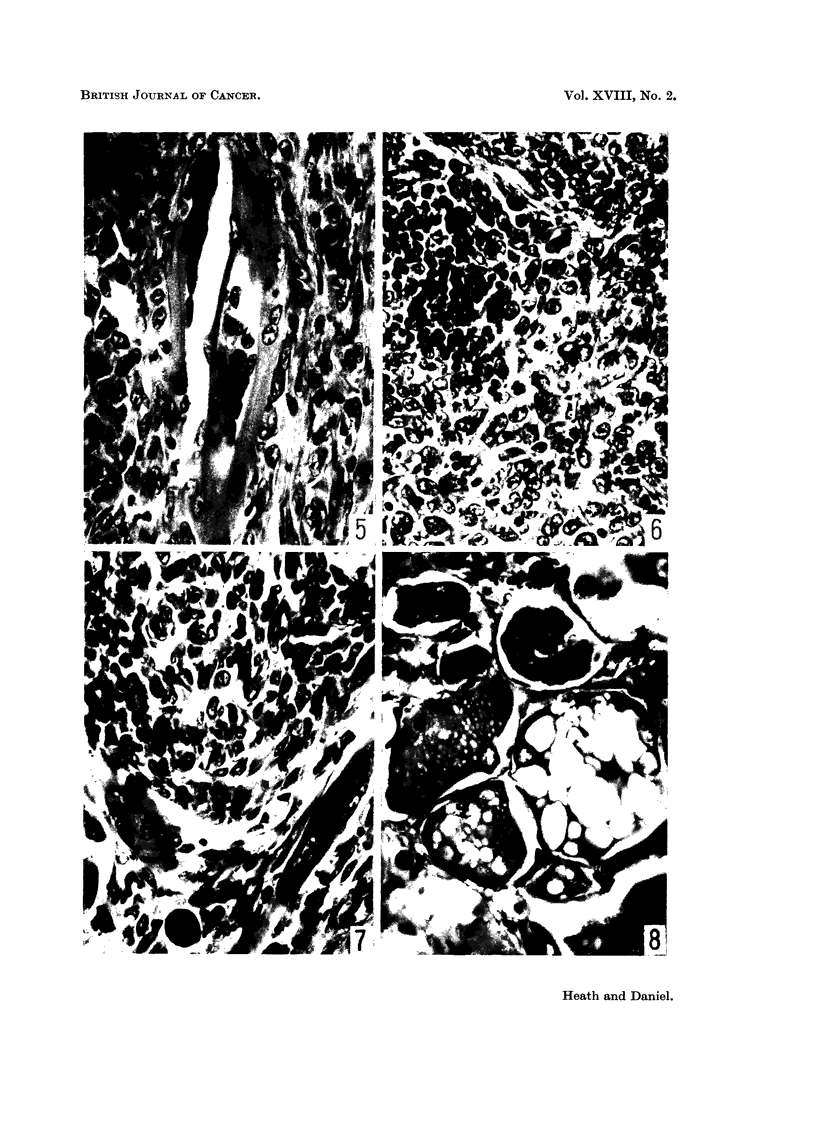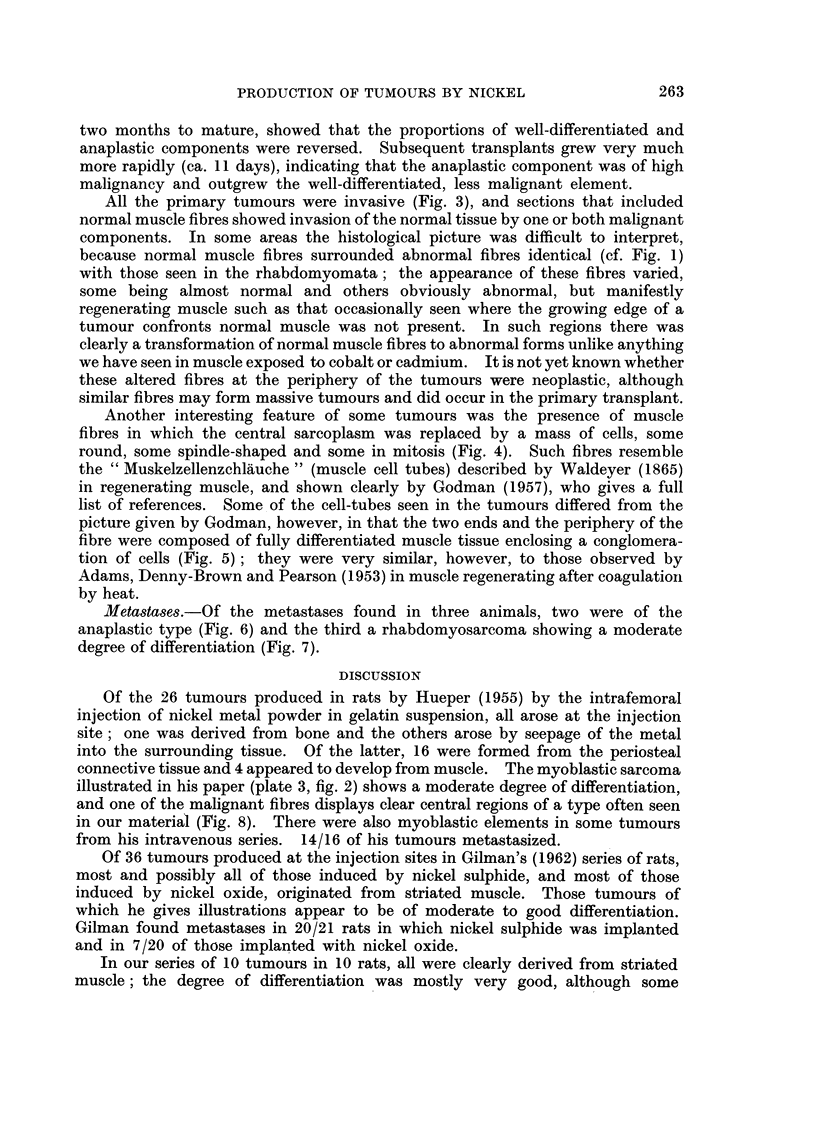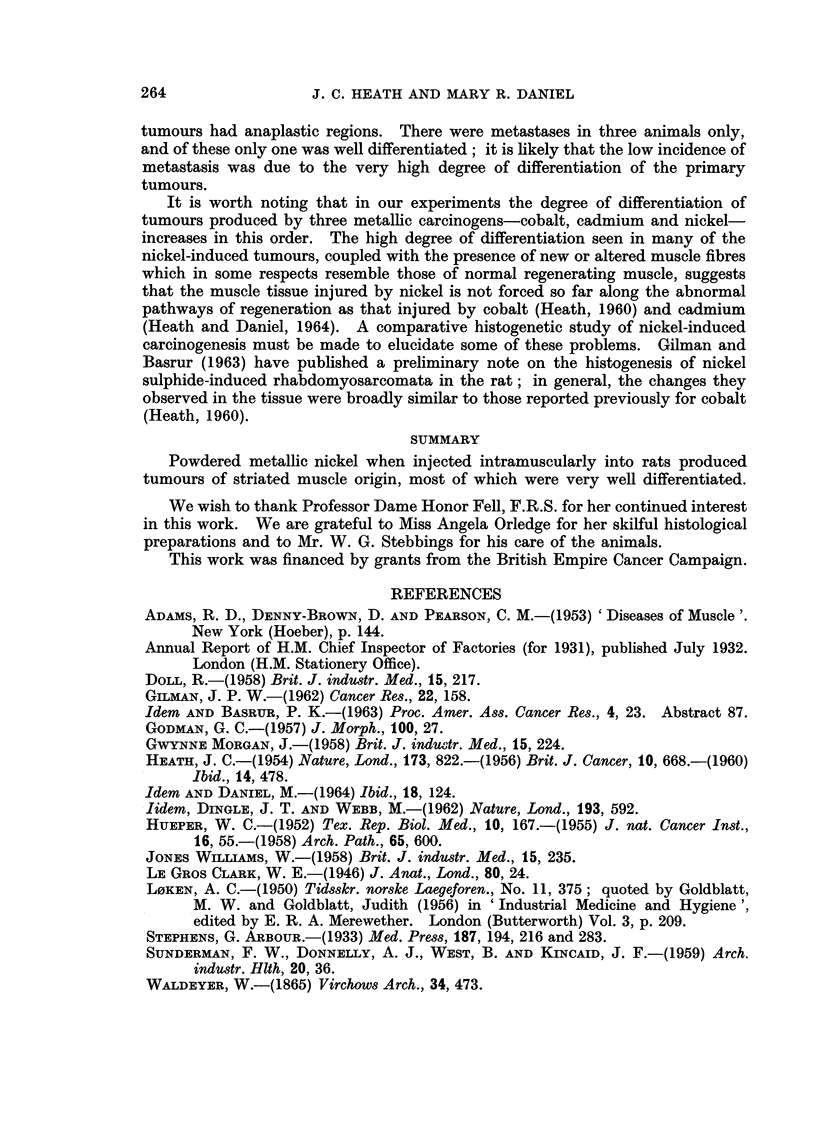# The Production of Malignant Tumours by Nickel in the Rat

**DOI:** 10.1038/bjc.1964.30

**Published:** 1964-06

**Authors:** J. C. Heath, Mary R. Daniel

## Abstract

**Images:**


### THE PRODUCTION OF MALIGNANT TUMOURS BY NICKEL IN THE RAT

J. C. HEATH AND MARY R. DANIEL
*From the Strangeways Research Laboratory, Cambridge*

Received for publication March 6, 1964

In May 1932 a Parliamentary question drew attention to a number of cases of nasal cancer which had occurred among workers in a nickel refinery at Clydach, South Wales. The report of the Chief Inspector of Factories for 1931 (published in July 1932) listed several cases, and Stephens (1933), in discussing cases of industrial epitheliomata, suggested that nickel was one of the agents responsible. It was already known that nickel compounds, including gaseous nickel carbonyl, were toxic, but they were not suspected of being carcinogens. Subsequent statistical investigations by Doll (1958) and Gwynne Morgan (1958), and pathological studies by Løken (1950) and Jones Williams (1958) have confirmed the relatively high incidence of respiratory cancer in nickel workers.

Animal experiments by Hueper (1958) on the effect of inhalation of metallic nickel powder by rats and guinea-pigs, and by Sunderman, Donnelly, West and Kincaid (1959) on the effect of inhalation of nickel carbonyl by rats, confirmed that nickel could produce neoplastic changes in the respiratory tract, leading sometimes to frankly malignant tumours (Sunderman *et al.*, 1959). Hueper (1952) had previously shown that powdered nickel, injected into rats by various routes, produced malignant tumours in at least 8 out of 70 animals. He also showed (Hueper, 1955) that the same agent, introduced by five different routes into rats, mice, and rabbits, produced malignant tumours of various types in 27 out of 100 rats, one out of six rabbits, and none of 125 mice. It is of interest that he draws attention to a possible species-specificity.

Recently, Gilman (1962) has shown that nickel sulphide and nickel oxide, injected intramuscularly into rats and mice, produce tumours at the injection site. The tumours he obtained in the rats were mainly rhabdomyosarcomata ; in the mice, although many of the tumours had the characteristics of fibrosarcomata, their cellularity, numerous oval nuclei and lack of collagen suggested to him that they contained myomatous elements. He has observed (personal communication) a strain-specificity of the carcinogenic action of nickel in rats.

We are investigating the mechanisms of metal carcinogenesis, and thought it necessary to look for the possible carcinogenic effects of pure nickel powder under the same conditions as those in which cobalt (Heath, 1954 and 1956) and cadmium (Heath, Daniel, Dingle and Webb, 1962 ; Heath and Daniel, 1964) are carcinogenic.

### MATERIALS AND METHODS

Ten female rats of the hooded strain, aged 2–3 months, were used. 0·0283 g. of spectrographically pure nickel metal powder (Johnson Matthey) was shaken into suspension with 0·4 ml. of fowl serum and injected into the muscle of the right thigh of each animal from the medial aspect, approximately parallel with the femur and directed towards the hip. On microscopical examination the metal powder was found to consist mainly of aggregates ranging from $3\ \mu \times 3\ \mu$ to $117\ \mu \times 87\ \mu$, composed of small, mostly spherical, particles of 0·5–0·8 $\mu$ diameter. There were a very few small single particles of about $1{\cdot}7\ \mu \times 2{\cdot}5\ \mu$; simple trituration of the larger aggregates with a glass rod in a little water did not easily sub-divide them further. The characteristics of this nickel sample were similar to those of the one obtained by Hueper (1955) from the International Nickel Co.

Control injections with fowl serum alone were not made, since in previous work (Heath, 1956), serum produced no reaction or tumours.

### RESULTS

There was no clinical evidence of an immediate response to the injection, either local or systemic.

All the animals developed tumours at the injection site. The earliest tumour was noticed 17 weeks after injection and was removed for histological examination at $18\frac{5}{7}$ weeks; most of the others appeared within the next 5 weeks and the last was taken for histological examination at $40\frac{3}{7}$ weeks.

Three animals showed metastases, all of which were in the prevertebral lymph nodes.

### *Gross appearance of tumours*

The major dimensions of the tumours ranged from $3 \times 2\frac{1}{2} \times 2\frac{1}{2}$ cm. to $5 \times 4 \times 4$ cm. Most of the tumours were pinkish-white, and the consistency varied from soft to firm; they showed very little necrosis, but three contained cystic regions filled with blood-stained fluid or thrombus.

### *Histological appearance of tumours*

*Primary tumours.*—All of the tumours had clearly originated in striated muscle tissue; seven were well differentiated, and in the others differentiation ranged from poor to moderate. In the best differentiated regions, the tissue consisted of interwoven bundles of striated muscle fibres (Fig. 1), the disorientation of which resembled that seen in sections of muscle regenerating after crush injury (Le Gros Clark, 1946). We propose to use the term "rhabdomyoma" for tumours with this appearance, without thereby implying that they are benign. A tumour having this rhabdomyomatous appearance in most regions, together with small interspersed anaplastic components (Fig. 2), was used successfully for serial transplantation; histological examination of the first transplant, which took

---

EXPLANATION OF PLATES

All stained with Azan. ×450

FIG. 1.—Area of tumour having an appearance defined as rhabdomyoma.
FIG. 2.—Anaplastic region of a similar tumour.
FIG. 3.—Invasion of tendon by fairly well differentiated rhabdomysarcoma.
FIG. 4.—" Muscle tube ", similar to those found in regenerating muscle, lying between two normal muscle fibres.
FIG. 5.—Muscle fibre in which the centre is occupied by discrete cells.
FIG. 6.—Lymph node almost replaced by anaplastic tumour tissue.
FIG. 7.—Lymph node almost replaced by well differentiated rhabdomysarcoma.
FIG. 8.—Cross-sections of rhabdomyomatous fibres showing varying degree of sarcoplasmic vacuolation.

Heath and Daniel.

Heath and Daniel.

two months to mature, showed that the proportions of well-differentiated and anaplastic components were reversed. Subsequent transplants grew very much more rapidly (ca. 11 days), indicating that the anaplastic component was of high malignancy and outgrew the well-differentiated, less malignant element.

All the primary tumours were invasive (Fig. 3), and sections that included normal muscle fibres showed invasion of the normal tissue by one or both malignant components. In some areas the histological picture was difficult to interpret, because normal muscle fibres surrounded abnormal fibres identical (cf. Fig. 1) with those seen in the rhabdomyomata ; the appearance of these fibres varied, some being almost normal and others obviously abnormal, but manifestly regenerating muscle such as that occasionally seen where the growing edge of a tumour confronts normal muscle was not present. In such regions there was clearly a transformation of normal muscle fibres to abnormal forms unlike anything we have seen in muscle exposed to cobalt or cadmium. It is not yet known whether these altered fibres at the periphery of the tumours were neoplastic, although similar fibres may form massive tumours and did occur in the primary transplant.

Another interesting feature of some tumours was the presence of muscle fibres in which the central sarcoplasm was replaced by a mass of cells, some round, some spindle-shaped and some in mitosis (Fig. 4). Such fibres resemble the " Muskelzellenzchläuche " (muscle cell tubes) described by Waldeyer (1865) in regenerating muscle, and shown clearly by Godman (1957), who gives a full list of references. Some of the cell-tubes seen in the tumours differed from the picture given by Godman, however, in that the two ends and the periphery of the fibre were composed of fully differentiated muscle tissue enclosing a conglomeration of cells (Fig. 5) ; they were very similar, however, to those observed by Adams, Denny-Brown and Pearson (1953) in muscle regenerating after coagulation by heat.

*Metastases.*—Of the metastases found in three animals, two were of the anaplastic type (Fig. 6) and the third a rhabdomyosarcoma showing a moderate degree of differentiation (Fig. 7).

### DISCUSSION

Of the 26 tumours produced in rats by Hueper (1955) by the intrafemoral injection of nickel metal powder in gelatin suspension, all arose at the injection site ; one was derived from bone and the others arose by seepage of the metal into the surrounding tissue. Of the latter, 16 were formed from the periosteal connective tissue and 4 appeared to develop from muscle. The myoblastic sarcoma illustrated in his paper (plate 3, fig. 2) shows a moderate degree of differentiation, and one of the malignant fibres displays clear central regions of a type often seen in our material (Fig. 8). There were also myoblastic elements in some tumours from his intravenous series. 14/16 of his tumours metastasized.

Of 36 tumours produced at the injection sites in Gilman's (1962) series of rats, most and possibly all of those induced by nickel sulphide, and most of those induced by nickel oxide, originated from striated muscle. Those tumours of which he gives illustrations appear to be of moderate to good differentiation. Gilman found metastases in 20/21 rats in which nickel sulphide was implanted and in 7/20 of those implanted with nickel oxide.

In our series of 10 tumours in 10 rats, all were clearly derived from striated muscle ; the degree of differentiation was mostly very good, although some tumours had anaplastic regions. There were metastases in three animals only, and of these only one was well differentiated ; it is likely that the low incidence of metastasis was due to the very high degree of differentiation of the primary tumours.

It is worth noting that in our experiments the degree of differentiation of tumours produced by three metallic carcinogens—cobalt, cadmium and nickel—increases in this order. The high degree of differentiation seen in many of the nickel-induced tumours, coupled with the presence of new or altered muscle fibres which in some respects resemble those of normal regenerating muscle, suggests that the muscle tissue injured by nickel is not forced so far along the abnormal pathways of regeneration as that injured by cobalt (Heath, 1960) and cadmium (Heath and Daniel, 1964). A comparative histogenetic study of nickel-induced carcinogenesis must be made to elucidate some of these problems. Gilman and Basrur (1963) have published a preliminary note on the histogenesis of nickel sulphide-induced rhabdomyosarcomata in the rat ; in general, the changes they observed in the tissue were broadly similar to those reported previously for cobalt (Heath, 1960).

### SUMMARY

Powdered metallic nickel when injected intramuscularly into rats produced tumours of striated muscle origin, most of which were very well differentiated.

We wish to thank Professor Dame Honor Fell, F.R.S. for her continued interest in this work. We are grateful to Miss Angela Orledge for her skilful histological preparations and to Mr. W. G. Stebbings for his care of the animals.

This work was financed by grants from the British Empire Cancer Campaign.

### REFERENCES

Adams, R. D., Denny-Brown, D. and Pearson, C. M.—(1953) ‘ Diseases of Muscle ’. New York (Hoeber), p. 144.

Annual Report of H.M. Chief Inspector of Factories (for 1931), published July 1932. London (H.M. Stationery Office).

Doll, R.—(1958) *Brit. J. industr. Med.*, **15**, 217.

Gilman, J. P. W.—(1962) *Cancer Res.*, **22**, 158.

*Idem* and Basrur, P. K.—(1963) *Proc. Amer. Ass. Cancer Res.*, **4**, 23. Abstract 87.

Godman, G. C.—(1957) *J. Morph.*, **100**, 27.

Gwynne Morgan, J.—(1958) *Brit. J. industr. Med.*, **15**, 224.

Heath, J. C.—(1954) *Nature, Lond.*, **173**, 822.—(1956) *Brit. J. Cancer*, **10**, 668.—(1960) *Ibid.*, **14**, 478.

*Idem* and Daniel, M.—(1964) *Ibid.*, **18**, 124.

*Iidem*, Dingle, J. T. and Webb, M.—(1962) *Nature, Lond.*, **193**, 592.

Hueper, W. C.—(1952) *Tex. Rep. Biol. Med.*, **10**, 167.—(1955) *J. nat. Cancer Inst.*, **16**, 55.—(1958) *Arch. Path.*, **65**, 600.

Jones Williams, W.—(1958) *Brit. J. industr. Med.*, **15**, 235.

Le Gros Clark, W. E.—(1946) *J. Anat., Lond.*, **80**, 24.

Løken, A. C.—(1950) *Tidsskr. norske Laegeforen.*, No. 11, 375 ; quoted by Goldblatt, M. W. and Goldblatt, Judith (1956) in ‘ Industrial Medicine and Hygiene ’, edited by E. R. A. Merewether. London (Butterworth) Vol. 3, p. 209.

Stephens, G. Arbour.—(1933) *Med. Press*, **187**, 194, 216 and 283.

Sunderman, F. W., Donnelly, A. J., West, B. and Kincaid, J. F.—(1959) *Arch. industr. Hlth*, **20**, 36.

Waldeyer, W.—(1865) *Virchows Arch.*, **34**, 473.